# Qingfei Formula Protects against Human Respiratory Syncytial Virus-induced Lung Inflammatory Injury by Regulating the MAPK Signaling Pathway

**DOI:** 10.2174/1386207326666230821121358

**Published:** 2024-04-26

**Authors:** Ya-Lei Sun, Pei-Pei Zhao, Cheng-Bi Zhu, Xin-Min Li, Bin Yuan

**Affiliations:** 1 Department of Pediatrics, Affiliated Hospital of Nanjing University of Chinese Medicine, Nanjing, 210000, China;; 2 Jiangsu Key Laboratory of Pediatric Respiratory Disease, Institute of Pediatrics, Affiliated Hospital of Nanjing University of Chinese Medicine, Nanjing, 210000, China;; 3 Henan University of Chinese Medicine, Zhengzhou, 450000, China

**Keywords:** Qingfei formula, human respiratory syncytial virus pneumonia, network pharmacology, molecular docking simulation, glycoprotein, lung inflammatory injury

## Abstract

**Objective:**

Qingfei formula (QF) is an empirical formula that shows good clinical efficacy in treating human respiratory syncytial virus pneumonia (RSVP). However, the underlying mechanism remains unclear. This study explores the possible pharmacological actions of QF in RSVP treatment.

**Methods:**

We used a network pharmacology approach to identify the active ingredients of QF, forecast possible therapeutic targets, and analyze biological processes and pathways. Molecular docking simulation was used to evaluate the binding capability of active ingredients and therapeutic targets. Finally, *in vivo* experiments confirmed the reliability of network pharmacology-based prediction of underlying mechanisms.

**Results:**

The study identified 92 potential therapeutic targets and corresponding 131 active ingredients. Enrichment analysis showed that QF downregulated the MAPK signaling pathway and suppressed the inflammatory injury to the lungs induced by the RSV virus. Molecular docking simulations demonstrated that the core active ingredients of QF could stably bind to genes associated with the MAPK signaling pathway. QF had a protective effect against pneumonia in RSV-infected mice. The QF group exhibited a significant reduction in the levels of inflammatory mediators, interleukin-6 (IL-6), interleukin-8 (CXCL8, IL-8), and P-STAT3, compared to the RSV-induced group. The QF group showed remarkably inhibited MAPK1+3(P-ERK1+2) and MAPK8(P-JNK) protein expression.

**Conclusion:**

The current study showed that QF downregulated the MAPK signaling pathway, which inhibited pulmonary inflammation triggered by RSV infection. This study recommends the appropriate use of QF in the clinical management of RSVP.

## 1 INTRODUCTION

The human respiratory syncytial virus (RSV) belongs to the genus *Pneumovirus* of the family Paramyxoviridae. It is a single-stranded negative-sense virus. The viral RNA encodes 10 subgenomic mRNAs and 11 proteins, and the genome is contained in a nucleocapsid enveloped in a lipoprotein [1]. The primary surface glycoprotein (G) enables virus attachment, and the fusion protein (F) mediates virus-cell fusion [2]. RSV is a major contributor to pediatric hospitalizations due to RSV pneumonia (bronchiolitis or interstitial pneumonia) in infants, young children, and the elderly. Moreover, RSV is a factor in the deterioration of health in adults [3]. Intensive research on the treatment and control of RSV has been conducted, but no vaccinations or specific medicines are available. In view of the resultant lung tissue damage caused by RSV infection, it is very important to find a more suitable therapeutic strategy.

The Qingfei formula (QF) is a commonly used TCM prescription improvised from the classical prescription of Maxing Shigan decoction. QF has been used to treat viral pneumonia, especially human respiratory syncytial virus pneumonia (RSVP), for more than 40 years [4]. It consists of *Ephedra sinica Stapf. (Mahuang), Prunus armeniaca L. (Xingren), Gypsum Fibrosum (Shigao), Lepidium virginicum L. (Tinglizi), Morus alba L. (Sangbaipi), Peucedanum praeruptorum Dunn. (Qianhu), Bombyx mori Linnaeus. (Jiangcan), Salvia miltiorrhiza Bunge. (Danshen), Reynoutria japonica Houtt. (Huzhang),* and *Bistorta officinalis Delarbre (Quanshen)*. QF was found to play a vital regulation role in the immune system and inflammation after RSV infection [4]. However, the underlying mechanism is still unclear, and this lack of knowledge limits its therapeutic use. As a new area of pharmacology, network pharmacology provides new methods for elucidating the multiple mechanisms of the actions of drugs by exploring the disease targets [5]. This study used network pharmacology and *in vivo* experimental verification to provide a preliminary determination of the mechanism of QF in RSVP treatment. Fig. (**1**) displays the flow chart of the study.

## 2 MATERIALS AND METHODS

### 2.1 Search for Potential Therapeutic Target

To seek the active ingredients of QF, we used the Traditional Chinese Medicine Systems Pharmacology Database and Analysis Platform (TCMSP, https://old.tcmsp-e.com/tcmsp.php) and Traditional Chinese Medicine Integrative Database (TCMID, http://www.megabionet.org/tcmid/) [5, 6]. The search terms were “Mahuang,” “Shigao,” “Xingren,” “Sangbaipi,” “Tinglizi,” “Qianhu,” “Huzhang,” “Quanshen,” “Jiangcan,” and “Danshen.” Oral bioavailability (OB) ≥ 0.3 and drug-likeness (DL) ≥ 0.18 were the screening conditions. In addition, we conducted a literature search. After eliminating duplicates, search results were merged to determine the active ingredient targets. Each target name was changed to match the official gene names in the UniProt database (https://www.uniprot.org/).

To search for RSVP targets, we used the GeneCards (https://www.genecards.org/), DisGeNET database (http://www.disgenet.org/), and Online Mendelian Inheritance in Man (OMIM, https://www.omim.org/) databases [7, 8]. The search terms were “RSV pneumonia,” “respiratory syncytial virus pneumonia,” and “RSV-induced lung inflammation.” After eliminating duplicates, retrieval results were merged to obtain the RSVP targets.

The interaction between QF targets and RSVP targets was examined using the R software to identify potential therapeutic targets [9].

### 2.2 GO and KEGG Analyses

We used the R package clusterProfiler for Gene Ontology (GO) function enrichment analysis and Kyoto Encyclopedia of Genes and Genomes (KEGG) pathway enrichment analysis to demonstrate the role of potential therapeutic targets in gene function and signaling pathways [10]. Following the completion of the GO function enrichment study, which comprised biological process (BP), cellular component (CC), and molecular function (MF) items, the top ten pertinent GO enrichment items were displayed as bubble plots. The top twenty pertinent KEGG pathways were presented as bubble plots.

### 2.3 Construction of Ingredient-Target Network

Our study thoroughly examined the molecular mechanism of QF in treating RSVP by creating an active ingredient-potential therapeutic target network diagram using Cytoscape 3.6.0 software [11]. The core nodes of the network were analyzed using the DegreeFilter plugin. The top five pharmaceutical ingredients (according to degree value) were considered the core active ingredients of QF.

### 2.4 Construction of Protein-protein Interaction Network

The STRING database (https://string-db.org) seeks to assemble and combine all publicly accessible sources of protein-protein interaction (PPI) knowledge to develop a comprehensive network [12]. To assess the interactions among the potential therapeutic targets, we uploaded the potential therapeutic targets and created a PPI network graph (interaction score = 0.7). Then, we loaded the PPI data into the Cytoscape 3.6.0 software to visualize results and used the CytoNCA plugin to determine the core genes.

### 2.5 Molecular Docking Simulation

Molecular docking was used to assess the binding between core genes and core active ingredients. Molecular docking simulations were performed following established protocols using AutoDock 4.2 and AutoDock Vina software [13].

### 2.6 Experimental Verification

#### 2.6.1 Drugs and Reagents

QF is a Chinese medicine formula consisting of 10 herbs, including 10 g Mahuang, 24 g Shigao, 10 g Xingren, 10 g Sangbaipi, 6 g Tinglizi, 10 g Qianhu, 12 g Huzhang, 12 g Quanshen, 6 g Jiangcan, and 6 g Danshen. All herbs were provided by Jiangsu Chinese Medicine Hospital. The concentration of the crude drug was 3.7 g/mL (about 3 times the clinical dose) [14]. The Wuhan University Institute of Viruses provided the human RSV strain; Proteintech (Wuhan, China) provided JNK and ERK1+2 antibodies. ABclonal (Wuhan, China) provided the IL-6 and P-STAT3 antibodies; Sichuan Baili Pharmaceutical Co., Ltd. provided ribavirin.

#### 2.6.2 Protocol of Animal Experiments In Vivo

Fifty specific pathogen-free grade BALB/c mice (20.0 ± 2.0 g; 6 - 8 weeks) were purchased from Jiangsu Qinglongshan Co., Ltd. (Jiangsu, China). The mice were randomly divided into 5 groups (n=10): the control group, the RSV-induced group (Model), the QF low-dose (QF-LD) group (0.2 mL/d), the QF high-dose (QF-HD) group (0.6 mL/d), and the ribavirin control group (Riba) (46 mg/kg/d). All mice, excluding those in the control group, were given an intranasal injection of the RSV virus (1.4 × 107 plaque-forming units) under a light anesthetic (isoflurane). Mice in the control group received the same volume of sodium chloride solution. The treatment groups received oral medications 48 hours after infection. Sodium chloride solution was administered to the control and model groups in equal amounts. The dosage continued for three days, and the mice were observed daily for health status. Mice were sacrificed 5 days after infection to collect pertinent samples [14]. All animal experiments were approved by the Institutional Animal Care and Use Committee of the Laboratory Animal Services Center at Nanjing University of Chinese Medicine (approval ID: SYXK (Su) 2018-0049) and performed according to the relevant guidelines and regulations.

#### 2.6.3 Pathological and Immunohistochemical Examination

After preservation with a 4% paraformaldehyde solution for 24 hours, mouse lung tissue was dehydrated. The mouse samples were divided into 3-mm thick sections after paraffin embedding. Hematoxylin and eosin (HE) staining was applied to the slices before microscopic examination in a double-blind fashion. The average of five fields in each section, rated from 0 to 3 (normal, mild, moderate, and severe), was used to reflect the degree of pathological alterations [15, 16]. Additional tissue samples were used from each group for immunohistochemistry tests, followed by P-STAT3 expression analysis. The expression of the favorable outcomes was cytoplasmic brown staining. The study used Image-Pro Plus 6.0 software to calculate the average integrated optical density (IOD) [17].

#### 2.6.4 Immunofluorescence Assay

Lung tissues were deparaffinized and dehydrated. Following antigen retrieval, lung slices were processed for immunofluorescence. The fixed tissue samples were placed on the cover glass, blocked with donkey serum (Solarbio, Beijing, China), and probed with RSV-antibody [18]. The fixed tissue sections were rinsed thrice with PBS before incubation with the secondary antibody at 37°C for 50 min while keeping out of the light. The cell nuclei were stained with 4′,6-diamidino-2-phenylindole at 37°C for 10 min in the dark. Finally, an anti-fade mounting buffer was applied to the sectioned tissues for fluorescence microscopy (Olympus).

#### 2.6.5 Western Blot Analysis

Western blot analysis was performed following the standard procedure [19]. Equal amounts of protein were separated using 10% SDS PAGE and then transferred to a PVDF membrane (Merck Millipore, IRL). The membrane was treated with the primary antibody overnight at 4°C, followed by an hour-long incubation with the corresponding secondary antibody at room temperature to block non-specific binding sites. Antigen-antibody complexes were discovered using the ECL reagent (Yeasen, Shanghai, China).

#### 2.6.6 Statistical Analysis

GraphPad Prism 5.0 was used for the statistical analysis. All data have been reported as mean ± standard error and gathered from at least three different experiments. A comparison of the means of several groups was made using a one-way analysis of variance test. The levels of significance were specified at 0.05 and 0.01.

## 3 RESULTS

### 3.1 Identification of Potential Therapeutic Targets

We combined and deduplicated the search results after filtering them based on the herbal names and screening conditions. The following were the active ingredients in QF, according to the results: 23 species of Mahuang, 1 species of Shigao, 19 species of Xingren, 32 species of Sangbaipi, 12 species of Tinglizi, 24 species of Qianhu, 10 species of Huzhang, 6 species of Quanshen, 2 species of Jiangcan, and 65 species of Danshen. After deleting 27 duplicate ingredients, we obtained 167 active ingredients. Five active ingredients in QF were obtained through a literature search. Further search for the active ingredient targets yielded a total of 247 targets.

**Fig. (2):**
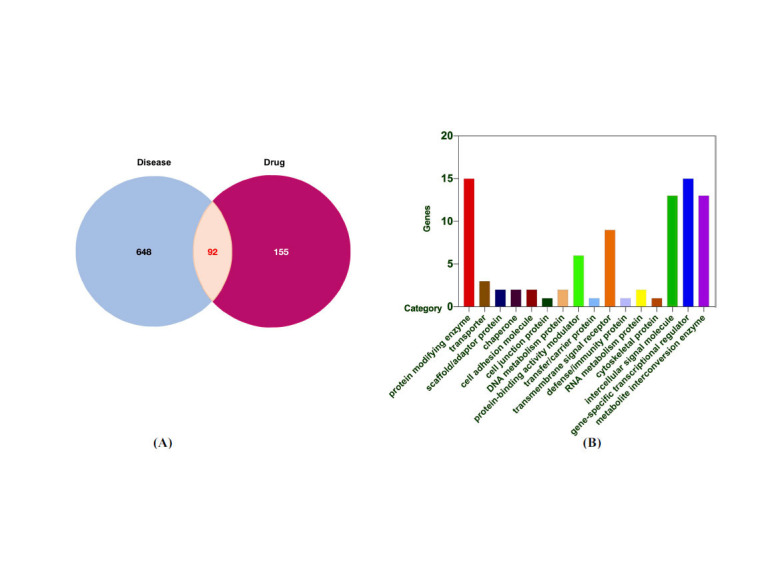
Prediction of the potential therapeutic targets. (**A**) Venn diagram of the potential therapeutic targets. (**B**) The classification of potential therapeutic targets according to their biochemical criteria.

We searched the GeneCards, DisGeNET, and OMIM databases and obtained 685 RSVP targets, 2 RSVP targets, and 71 RSVP targets. A total of 740 RSVP targets were obtained after integrating the outcomes of the three databases and eliminating the duplicates. The 247 QF targets and 740 RSVP targets were then overlapped to yield potential therapeutic targets. Ultimately, interaction analysis produced 92 potential therapeutic targets (Fig. **2A** and Table **1**). Then, potential therapeutic targets were classified according to their biochemical criteria, as shown in Fig. (**2B**). These 92 potential therapeutic targets mainly included gene-specific transcriptional regulators and protein-modifying enzymes.

### 3.2 Biological Function Analysis of Potential Therapeutic Targets

**Fig. (3):**
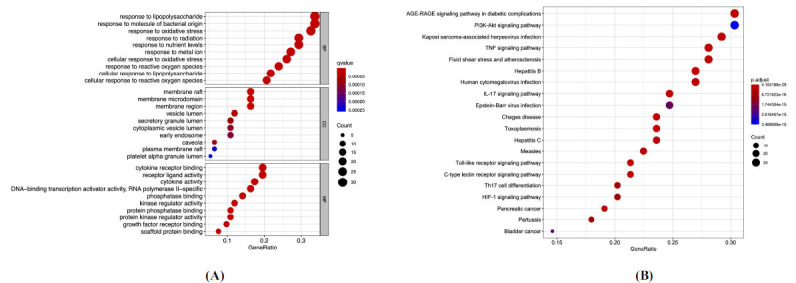
GO (**A**) and KEGG (**B**) enrichment analysis of the potential therapeutic targets.

GO function enrichment analysis showed the top ten GO items for BP, CC, and MF, and KEGG pathway enrichment analysis identified 20 KEGG pathways. GO functional enrichment results primarily identified a reaction to lipopolysaccharides, molecules of bacterial origin, and oxidative stress as BP items of potential therapeutic targets. Potential therapeutic targets for CC items mainly comprised membrane raft, membrane microdomain, and membrane region. Potential therapeutic targets for MF items comprised cytokine receptor binding, receptor-ligand activity, and cytokine activity. KEGG results showed AGE-RAGE signaling route, the PI3K-Akt signaling pathway, and Kaposi's sarcoma-associated herpesvirus infection signaling pathway to be the key signaling pathways linked with potential therapeutic targets (Fig. **3A** and **3B**).

### 3.3 Ingredient-Target Network

**Fig. (4):**
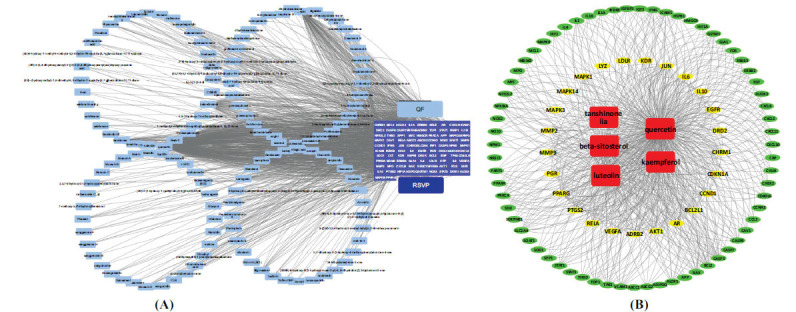
(**A**) Active ingredient-potential therapeutic target network. (**B**) Five core ingredients in potential therapeutic target network (red represents ingredients; yellow and green represent targets).

The ingredient-target network, depicted in Fig. (**4A**), was built to clarify the interactions between them. It had 223 nodes (92 potential therapeutic targets and corresponding 131 active ingredients). The nodes had different colors, with blue representing potential therapeutic targets and light blue representing the active ingredients. The edges were used to indicate the correlation between the nodes. The network analysis revealed that the average degree value for the active ingredients was 9.08, suggesting that QF has several targets for treating RSVP. Notably, the network contained five active ingredients with degrees ≧ 25, and these five ingredients considered to be the core active ingredients of QF were quercetin (degree = 416), luteolin (degree = 96), kaempferol (degree = 68), beta-sitosterol (degree = 61), and tanshinone (degree = 30) (Fig. **4B**).

### 3.4 PPI Network Analysis

**Fig. (5):**
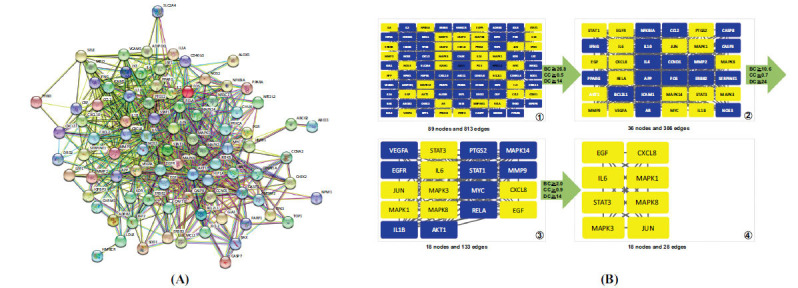
Protein-protein interaction network analysis. (**A**) PPI network. (**B**) The screening process of core genes.

For PPI network development and analysis, 92 potential therapeutic targets were loaded into the STRING database, and 813 edges reflecting the interaction between proteins (interaction score = 0.7) were produced by the network's 89 interacting targets (SOAT1, DUOX2, and GSTM1 were not involved in protein interaction) (Fig. **5A**). Based on three key parameters, betweenness centrality (BC), closeness centrality (CC), and degree centrality (DC), the topological feature analysis of the PPI network chose targets above median values as the core genes of prospective therapeutic targets. The first screening's threshold values were BC = 26.8, CC = 0.5, and DC = 14, and 36 nodes and 386 edges were the final results. The key genes, including EGF, MAPK1 (ERK2), MAPK3 (ERK1), MAPK8 (JNK1), JUN, STAT3, IL-6, and CXCL8 (IL8), were discovered after four screenings (Fig. **5B**).

### 3.5 Molecular Docking and Analysis

Molecular docking was performed to simulate the binding properties of various ingredients and key genes. The screening results mentioned above allowed for verifying EGF, MAPK1, MAPK3, MAPK8, JUN, STAT3, IL-6, and CXCL8. In Autodock, the 3D structure was imported and docked with various compounds, including quercetin, luteolin, kaempferol, beta-sitosterol, and tanshinone. Fig. (**6A**) shows the energy values of the chemicals used in the docking results. Fig. (**6B**) depicts the molecular docking pattern of tanshinone with MAPK3. These low docking energy values suggested that the ingredients might stably bind to the genes. Indirect evidence from the data further validates the validity of the network pharmacology prediction target by demonstrating agreement between the molecular docking and network pharmacology screening results.

### 3.6 Experimental Validation

#### 3.6.1 QF Mitigated RSV-induced Lung Injury and Inhibited Virus Replication

**Fig. (7):**
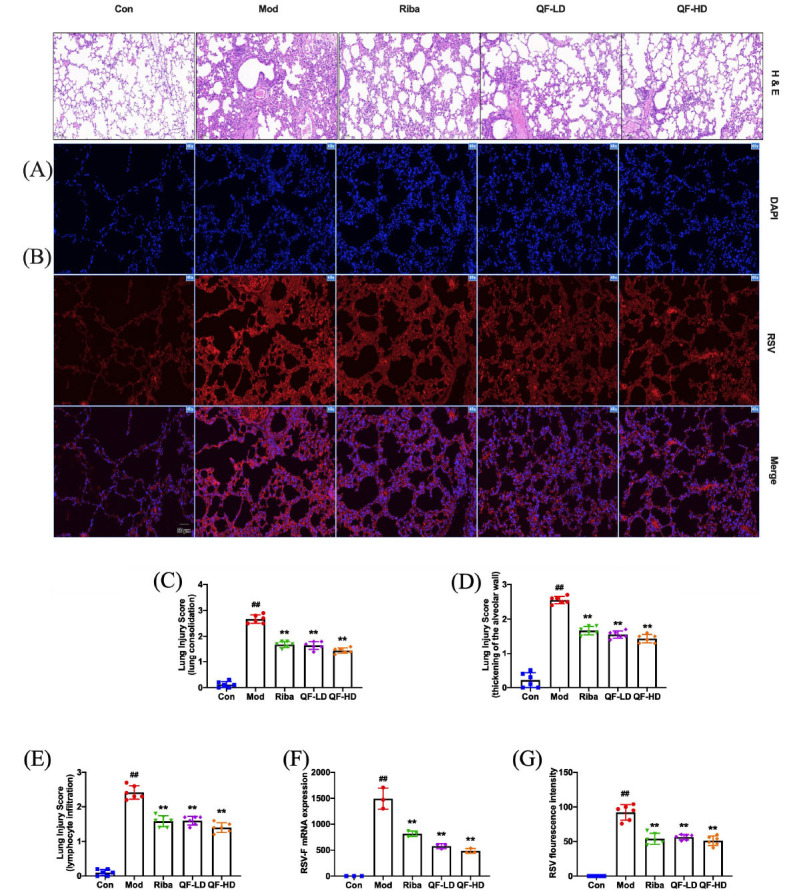
QF mitigated RSV-induced pulmonary histopathological damage and inhibited virus replication. (**A**) Pathological changes in lung tissue induced by RSV. Scale bar, 100 μm. (**B**) RSV expression assessed *via* an immunofluorescence assay. Scale bar, 50 μm. (**C-E**) Lung injury scores according to the degree of lung damage. (**F**) RSV-F mRNA level. (**G**) RSV fluorescence intensity. Data are presented as mean ± standard error. Significance: ## *P* <0.01 *vs.* control group; # *P* <0.05 *vs.* control group; ** *P* <0.01 *vs.* model group; * *P* <0.05 *vs.* model group.

H&E staining was used to examine the histological alterations in the lung tissue. According to Fig. (**7A**), mice lung tissue in the control group showed distinct alveolar lobules and alveolar cavities without leakage or cell infiltration in the alveolar gaps or the interstitium. RSV infection resulted in severe pulmonary inflammation characterized by lung consolidation, thickening of the alveolar wall, and lymphocytic infiltration. Inflammatory cells also invaded the alveolar space and lung interstitium due to the RSV infection. Compared to the model group, the QF-treated groups showed significantly lower scores for pathological damage and lung injury (lung consolidation, thickening of the alveolar wall, and lymphocyte infiltration), with mild inflammatory cell infiltration and protein leakage in the alveolar cavity (Fig. **7C-E**).

The severity of RSV infection is associated with the level of virus amplification. The relative expression of RSV-F genes was detected using QPCR to confirm RSV virus replication in the lung. As shown in Fig. (**7F**), RSV infection caused a marked increase in RSV-F mRNA levels in mouse lung tissue. Compared to the model group, the RSV-F mRNA levels in the QF-treated groups showed a significant dose-dependent reduction. We used an immunofluorescence technique to measure the virus levels in the mouse lung and track the challenge dosage of the virus (Fig. **7B**). Quantitative analysis showed that the amount of virus in the QF-treated groups was much lower than in the model group (Fig. **7G**). Overall, these findings showed that, in RSV-infected mice, QF efficiently reduced lung damage and prevented virus multiplication.

#### 3.6.2 Effects of QF on Potential Targets

**Fig. (8):**
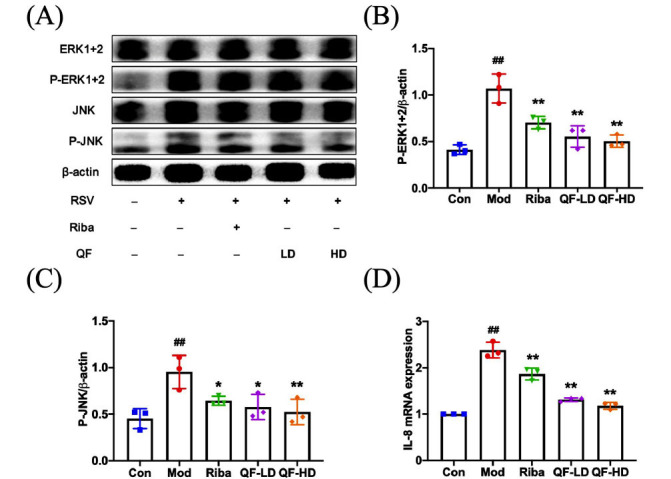
QF downregulated the protein levels of P-ERK1+2 and P-JNK and reduced mRNA levels of IL-8 in the lung tissues of RSV-challenged mice. (**A**) WB determination of P-ERK1+2 and P-JNK protein expression. (**B-C**) WB quantification of P-ERK1+2 and P-JNK. (**D**) mRNA expression of IL-8 was determined by real-time PCR. Data are presented as mean ± standard error. Significance: ## *P* <0.01 *vs.* control group; # *P* <0.05 *vs.* control group; ** *P* <0.01 *vs.* model group; * *P* <0.05 *vs.* model group.

We used Western blotting to assess the expression of proteins involved in the MAPK pathway to confirm the outcomes of network pharmacology and investigate the significance of prospective therapeutic targets. Results showed that P-ERK(1+2) and P-JNK expression levels in the model group were significantly higher than in the control group, proving that RSV might activate the MAPK signaling pathway. Compared to the model group, QF and ribavirin significantly decreased P-ERK(1+2) and P-JNK levels after RSV infection (Fig. **8A-C**). These findings imply that QF reduces RSV-induced inflammation by preventing MAPK signal activation.

**Fig. (9):**
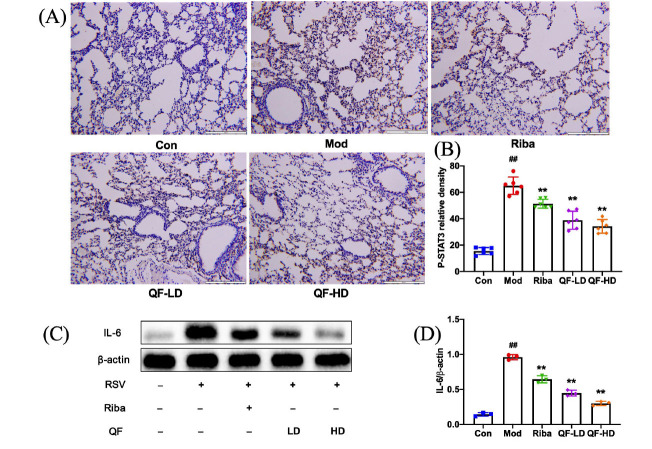
QF inhibited P-STAT3 and IL-6 protein expression in the lung tissues of RSV-induced mice. (**A**) IHC images displaying the protein levels of P-STAT3 in lung tissues. Scale bar, 200 μm. (**B**) Protein expression of P-STAT3 quantified using Image-Pro Plus 6.0 software. (**C**) WB determination of protein expression of IL-6. (**D**) WB quantification of IL6. Data are presented as mean ± standard error. Significance: ## *P* <0.01 *vs.* control group; # *P* <0.05 *vs.* control group; ** *P* <0.01 *vs.* model group; * *P* <0.05 *vs.* model group.

We assessed the level of inflammation-related genes in the MAPK signaling pathway to confirm the abovementioned finding. We conducted immunohistochemistry tests on mouse lung tissues. The outcomes demonstrated that the RSV model mice lung tissue had higher levels of P-STAT3 protein expression than the control group. Compared to the model group, the protein expression of P-STAT3 in lung tissue was significantly lower in the QF-treated groups (Fig. **9A** and **B**). We also assessed IL-6 protein concentrations in the lung. Compared to the model group, the protein expression of IL-6 in the QF-treated groups dramatically decreased (Fig. **9C** and **D**). The same pattern was seen in IL-8 mRNA expression levels (Fig. **8D**).

## 4 DISCUSSION

RSV-related lower respiratory tract infections are a substantial cause of death in young children, with over 200,000 occurrences per year worldwide [20]. RSV infections repeat throughout adulthood because an organism does not acquire long-lasting immunity against the infection. RSV infection is also common in elderly and immunosuppressed people [21]. A previous study on the elderly in the United States found that RSVP imposes a significant medical and economic burden on the elderly: 14,000 to 62,000 cases of RSV-related pneumonia hospitalization occur every year, with an estimated cost of $150 to $680 million [22]. Despite significant morbidity, there are currently no efficient vaccinations. The US Food and Drug Administration has approved the antiviral medications ribavirin and palivizumab for treating severe RSV infections. However, the use of these medications is limited by the significant risk of toxicity associated with ribavirin and high palivizumab cost [23].

Given these limitations, Chinese herbal medicine applications need to be promoted. In a previous multicenter clinical study, QF exhibited remarkable therapeutic effects on viral pneumonia, especially RSVP [24]. Specifically, compared to ribavirin, QF shortened the body temperature recovery time and significantly improved cough, copious sputum, and shortness of breath symptoms in patients [25]. QF dramatically decreased the exudation of inflammatory mediators (IL-6, 8) in mice with RSV-induced viral pneumonia and restored the Th1/Th2 imbalance, according to *in vivo* investigations [26–28].

This study used network pharmacology to investigate the mechanism of action of QF in treating RSVP. Molecular docking was used concurrently to simulate the binding properties of core active ingredients and core genes. Finally, *in vivo* test outcomes were obtained. These findings will be a foundation for future studies on RSVP treatment with QF.

We used a network pharmacology technique to discover 131 active components and 92 possible therapeutic targets. The top five active ingredients, quercetin, luteolin, kaempferol, beta-sitosterol, and tanshinone, were the core active ingredients in QF with the strongest correlations, according to the degree value ranking in the active ingredient-potential therapeutic target network. Reports show that some of these active substances may directly combat pneumonia viruses by reducing inflammation. Quercetin dramatically decreased the lung inflammation and mortality caused by LPS in mice and prevented the release of serum necrosis factor, interleukin-1, and interleukin-6 [29]. Luteolin and kaempferol are potential protective antagonists of acute lung injury in mice because they can suppress the activation of MAPK and NFκB pathways triggered by LPS [30, 31]. Tanshinone prevents inflammation and apoptosis and reduces acute lung injury caused by LPS in mice; β-sitosterol improves influenza symptoms. Virus-mediated recruitment of pathogenic cytotoxic T cells and immunological dysregulation can protect mice against deadly influenza A virus infection [32, 33]. These findings demonstrate the validity and viability of the network pharmacology approach to the search for active substances. Future studies should focus on the potential of active components to produce direct antiviral or anti-inflammatory effects.

Enrichment analysis of 92 prospective therapeutic targets showed that QF regulated the AGE-RAGE signaling pathway, the PI3K-Akt signaling system, and the herpesvirus infection linked to Kaposi sarcoma *in vivo*. According to the PPI network's topological feature analysis findings, QF could inhibit the MAPK signaling pathway to prevent the overexpression of inflammatory mediators in RSV-infected mice. The three main signaling routes (AGE-RAGE signaling pathway, PI3K-Akt signaling pathway, and Kaposi's sarcoma-associated herpesvirus infection) are strongly related to the MAPK signaling pathway [34-36]. Thus, to support this theory, we conducted *in vivo* tests.

Histopathological sections revealed lung consolidation, thickening of the alveolar wall, and lymphocytic infiltration in lung tissues of the model group mice. PCR results showed a significant increase in the RSV virus mRNA levels of the model group mice. However, the QF groups (LD and HD) exhibited a significant improvement in lung tissue injury and RSV virus mRNA levels in the lung. The results of the immunofluorescence assay were similar to PCR results. We also investigated whether the anti-inflammatory effects of QEOL involve the MAPK signaling pathway in the lung. Reports suggested that the MAPK signaling pathway showed significant activation during RSV infection in human alveolar basal epithelial cells [37]. Inflammation was found to be greatly influenced by the MAPK signaling pathway [38]. The p38 MAPK, ERK, and JNK pathways are interconnected MAPK pathways. Viral infection is one of the many intracellular and extracellular triggers that can activate these serine/threonine protein kinases. Several downstream transcription factors essential in controlling inflammation, including STAT-3, IL-6, and IL-8, are subsequently activated by p38 MAPK, JNK, and ERK [39-44]. The findings have demonstrated a considerable increase in the expression of P-ERK1+2 and P-JNK proteins in the lung tissue of the model group mice, indicating the activation of the MAPK signaling pathway. Inflammatory factors, such as IL-6, IL-8, and P-STAT3, have been found to be overexpressed concurrently with the MAPK signaling pathway activation. Oral QF therapy prevented P-ERK1+2 and P-JNK protein overexpression and decreased IL-6, IL-8, and STAT3 levels. These results revealed that QF controlled the MAPK signaling pathway to prevent excessive inflammatory responses. The outcomes further confirmed the dependability of the network pharmacology prediction target.

## CONCLUSION

The network pharmacological analysis demonstrated QF to be a complex preparation with multi-ingredient and multi-target properties, identifying 131 active components and 92 possible therapeutic targets linked to RSV virus infection. By controlling several targets, primarily those related to quercetin, luteolin, kaempferol, beta-sitosterol, and tanshinone, QF exerted therapeutic effects on RSVP. After the enrichment analysis of 92 potential therapeutic targets and topological feature analysis of the PPI network, we found that the MAPK signaling pathway plays an important role in the anti-inflammatory effects of QF in treating RSVP. Furthermore, molecular docking simulation confirmed genes related to the MAPK pathway, including EGF, MAPK1, MAPK3, MAPK8, JUN, STAT3, IL-6, and CXCL8, to exhibit good binding affinities with the corresponding active ingredients. According to the core gene screening results, we verified the effect of QF on RSV-infected mice and determined that QF exerts anti-inflammatory effects by downregulating the MAPK signaling pathway. *In vivo* experiment results further confirmed the reliability of network pharmacology to predict potential therapeutic targets. These findings offer a fresh approach to RSVP treatment and details on the mechanisms of QF.

## Figures and Tables

**Fig. (1):**
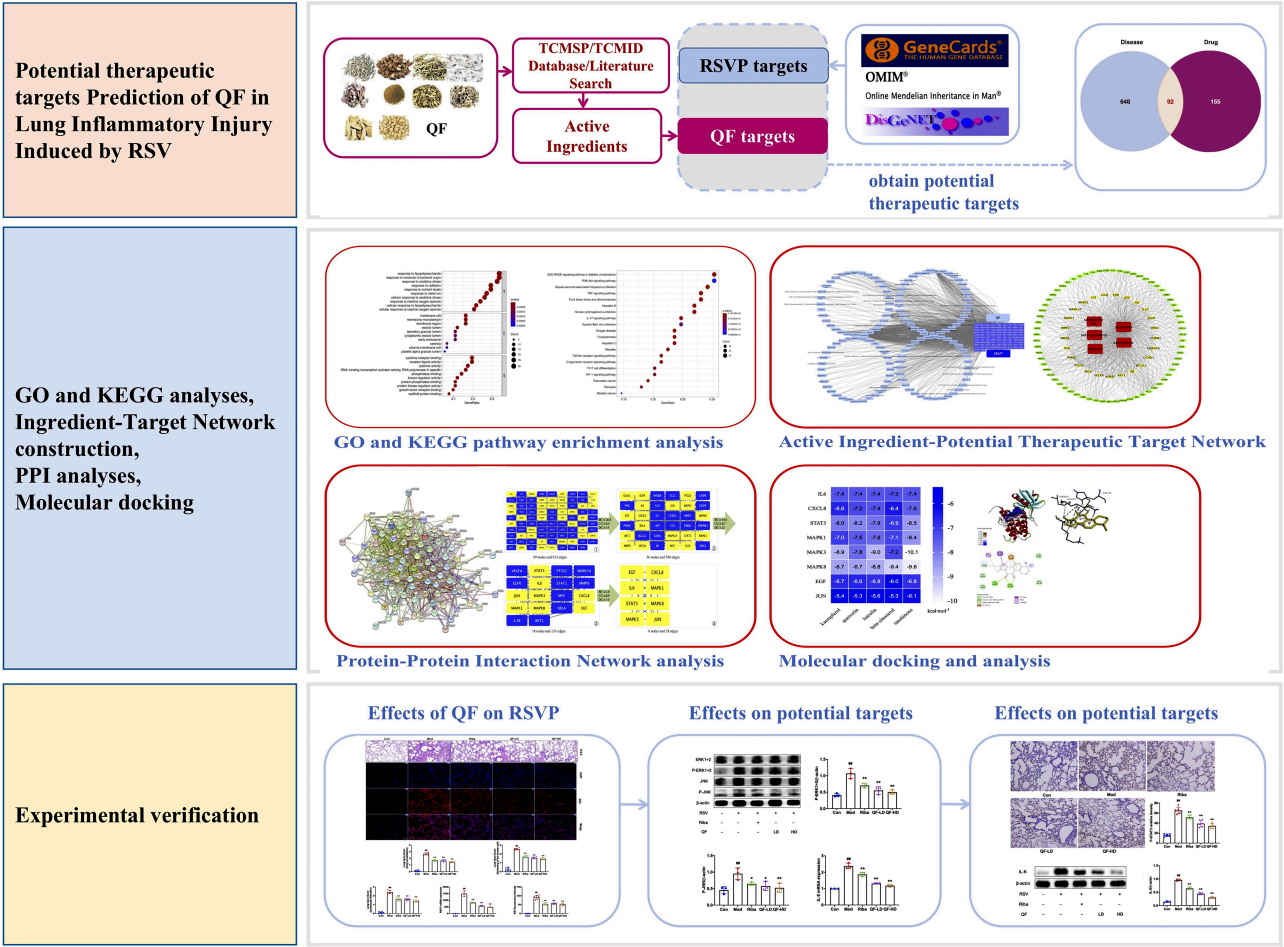
The flow chart of this study.

**Fig. (6):**
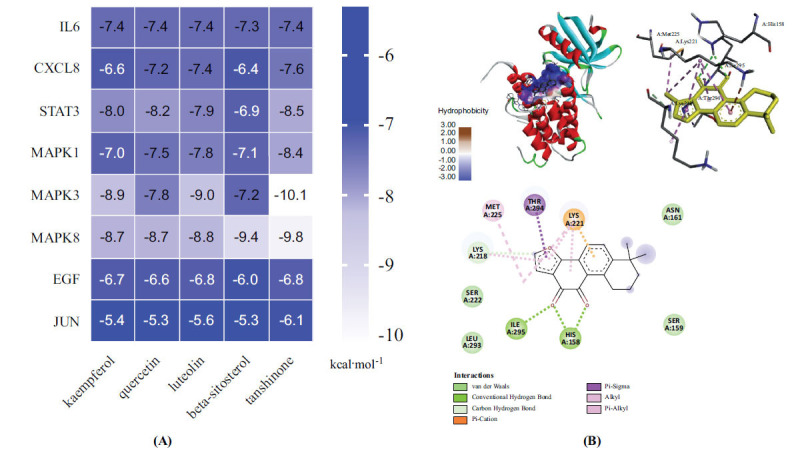
Molecular docking and analysis. (**A**) Heat map of the docking energy value. (**B**) Molecular docking pattern of tanshinone with MAPK3.

**Table 1:** 92 potential therapeutic targets.

**Mol. Id**	**Mol. Name**	**Target**	**Symbol**
MOL002222	Sugiol	Muscarinic acetylcholine receptor M1	CHRM1
MOL001601	1,2,5,6-tetrahydrotanshinone	Prostaglandin G/H synthase 2	PTGS2
MOL001601	1,2,5,6-tetrahydrotanshinone	Beta-2 adrenergic receptor	ADRB2
MOL001659	Poriferasterol	Progesterone receptor	PGR
MOL002222	Sugiol	D(2) dopamine receptor	DRD2
MOL002651	Dehydrotanshinone II A	Androgen receptor	AR
MOL002651	Dehydrotanshinone II A	Peroxisome proliferator activated receptor gamma	PPARG
MOL000006	Luteolin	Transcription factor p65	RELA
MOL000006	Luteolin	Epidermal growth factor receptor	EGFR
MOL000006	Luteolin	RAC-alpha serine/threonine-protein kinase	AKT1
MOL012753	Sanggenone F	Vascular endothelial growth factor receptor 2	KDR
MOL000098	Quercetin	Vascular endothelial growth factor A	VEGFA
MOL000098	Quercetin	G1/S-specific cyclin-D1	CCND1
MOL000098	Quercetin	Bcl-2-like protein 1	BCL2L1
MOL000098	Quercetin	Cyclin-dependent kinase inhibitor 1	CDKN1A
MOL000098	Quercetin	72 kDa type IV collagenase	MMP2
MOL000098	Quercetin	Matrix metalloproteinase-9	MMP9
MOL000098	Quercetin	Mitogen-activated protein kinase 1	MAPK1
MOL000098	Quercetin	Interleukin-10	IL10
MOL000098	Quercetin	Transcription factor AP-1	JUN
MOL000098	Quercetin	Caspase-3	CASP3
MOL000098	Quercetin	Cellular tumor antigen p53	TP63
MOL007154	Tanshinone iia	NF-kappa-B inhibitor alpha	NFKBIA
MOL000098	Quercetin	DNA topoisomerase 1	TOP1
MOL000006	Luteolin	E3 ubiquitin-protein ligase Mdm2	MDM2
MOL007088	Cryptotanshinone	Amyloid beta A4 protein	APP
MOL000006	Luteolin	Receptor tyrosine-protein kinase erbB-2	ERBB2
MOL000006	Luteolin	Caspase-7	CASP7
MOL000006	Luteolin	Intercellular adhesion molecule 1	ICAM1
MOL000006	Luteolin	Induced myeloid leukemia cell differentiation protein Mcl-1	MCL1
MOL000006	Luteolin	Interleukin-2	IL2
MOL000006	Luteolin	Interferon gamma	IFNG
MOL000006	Luteolin	Interleukin-4	IL4
MOL000006	Luteolin	Solute carrier family 2, facilitated glucose transporter member 4	SLC2A4
MOL000006	Luteolin	CD40 ligand	CD40LG
MOL012735	Mulberroside C_qt	Cyclin-A2	CCNA2
MOL012753	Sanggenone F	Nitric oxide synthase, inducible	NOS2
MOL003758	Iristectorigenin (9CI)	Mitogen-activated protein kinase 14	MAPK14
MOL001004	Pelargonidin	Glucocorticoid receptor	NR3C1
MOL007088	Cryptotanshinone	Signal transducer and activator of transcription 3	STAT3
MOL007154	Tanshinone iia	Apoptosis regulator Bcl-2	BCL2
MOL007154	Tanshinone iia	Proto-oncogene c-Fos	FOS
MOL007154	Tanshinone iia	Myc proto-oncogene protein	MYC
MOL007154	Tanshinone iia	Nucleophosmin	NPM1
MOL000358	Beta-sitosterol	Apoptosis regulator BAX	BAX
MOL000358	Beta-sitosterol	Caspase-8	CASP8
MOL000358	Beta-sitosterol	Protein kinase C alpha type	PRKCA
MOL000098	Quercetin	Pro-epidermal growth factor	EGF
MOL000098	Quercetin	Superoxide dismutase [Cu-Zn]	SOD1
MOL000098	Quercetin	Hypoxia-inducible factor 1-alpha	HIF1A
MOL000098	Quercetin	Signal transducer and activator of transcription 1-alpha/beta	STAT1
MOL000098	Quercetin	Caveolin-1	CAV1
MOL000098	Quercetin	Gap junction alpha-1 protein	GJA1
MOL000098	Quercetin	Interleukin-1 beta	IL1B
MOL000098	Quercetin	C-C motif chemokine 2	CCL2
MOL000098	Quercetin	E-selectin	SELE
MOL000098	Quercetin	Vascular cell adhesion protein 1	VCAM1
MOL000098	Quercetin	Interleukin-8	CXCL8
MOL000098	Quercetin	Dual oxidase 2	DUOX2
MOL000098	Quercetin	Nitric oxide synthase, endothelial	NOS3
MOL000098	Quercetin	Heat shock protein beta-1	HSPB1
MOL000098	Quercetin	Thrombomodulin	THBD
MOL000098	Quercetin	Plasminogen activator inhibitor 1	SERPINE1
MOL000098	Quercetin	Arachidonate 5-lipoxygenase	ALOX5
MOL000098	Quercetin	Interleukin-1 alpha	IL1A
MOL000098	Quercetin	Myeloperoxidase	MPO
MOL000098	Quercetin	ATP-binding cassette sub-family G member 2	ABCG2
MOL000098	Quercetin	Nuclear factor erythroid 2-related factor 2	NFE2L2
MOL000098	Quercetin	Poly [ADP-ribose] polymerase 1	PARP1
MOL000098	Quercetin	C-X-C motif chemokine 11	CXCL11
MOL000098	Quercetin	C-X-C motif chemokine 2	CXCL2
MOL000098	Quercetin	Serine/threonine-protein kinase Chk2	CHEK2
MOL000098	Quercetin	Peroxisome proliferator-activated receptor alpha	PPARA
MOL000098	Quercetin	C-reactive protein	CRP
MOL000098	Quercetin	C-X-C motif chemokine 10	CXCL10
MOL000098	Quercetin	Inhibitor of nuclear factor kappa-B kinase subunit alpha	CHUK
MOL000098	Quercetin	Osteopontin	SPP1
MOL000098	Quercetin	Insulin-like growth factor-binding protein 3	IGFBP3
MOL000098	Quercetin	Insulin-like growth factor II	IGF2
MOL000098	Quercetin	Interferon regulatory factor 1	IRF1
MOL000098	Quercetin	Receptor tyrosine-protein kinase erbB-3	ERBB3
MOL000098	Quercetin	Glutathione S-transferase Mu 1	GSTM1
MOL000422	Kaempferol	Inhibitor of nuclear factor kappa-B kinase subunit beta	IKBKB
MOL000422	Kaempferol	Mitogen-activated protein kinase 8	MAPK8
MOL004328	Naringenin	Mitogen-activated protein kinase 3	MAPK3
MOL004328	Naringenin	Low-density lipoprotein receptor	LDLR
MOL004328	Naringenin	3-hydroxy-3-methylglutaryl-coenzyme A reductase	HMGCR
MOL004328	Naringenin	Multidrug resistance-associated protein 1	ABCC1
MOL004328	Naringenin	Adiponectin	ADIPOQ
MOL004328	Naringenin	Sterol O-acyltransferase 1	SOAT1
MOL000098	Quercetin	Interleukin-6	IL6
MOL000296	Hederagenin	Lysozyme	LYZ

## Data Availability

The data supporting the findings of this study are available from the corresponding author [B.Y.] upon reasonable request.

## LIST OF ABBREVIATIONS

RSV: Respiratory Syncytial Virus
QF: Qingfei Formula
RSVP: Respiratory Syncytial Virus Pneumonia
TCMSP: Traditional Chinese Medicine Systems Pharmacology Database and Analysis Platform
TCMID: Traditional Chinese Medicine Integrative Database
OB: Oral Bioavailability
DL: Drug-Likeness
GO: Gene Ontology
KEGG: Kyoto Encyclopedia of Genes and Genomes
BP: Biological Process
CC: Cellular Component
MF: Molecular Function
